# Digestive tract reconstruction after laparoscopic proximal gastrectomy for Gastric cancer: A systematic review

**DOI:** 10.7150/jca.87315

**Published:** 2023-09-25

**Authors:** Li Li, Xufan Cai, Zhenghui Liu, Yiping Mou, YuanYu Wang

**Affiliations:** 1 General Surgery, Cancer Center, Department of Gastrointestinal and Pancreatic Surgery, Zhejiang Provincial People's Hospital (Affiliated People's Hospital), Hangzhou Medical College, Hangzhou, Zhejiang, China. Key Laboratory of Gastroenterology of Zhejiang Province, Hangzhou, Zhejiang, China.; 2 Zhejiang Chinese Medical University, Hangzhou, Zhejiang, China.

**Keywords:** gastroesophageal junction adenocarcinoma, proximal gastrectomy, adenocarcinoma, tubular gastroesophageal anastomosis, muscle flap anastomosis, jejunal interposition, double-tract reconstruction

## Abstract

The incidence of gastroesophageal junction adenocarcinoma has gradually increased. Proximal gastrectomy or total gastrectomy is recommended for early gastric cancer of the upper third of the stomach. Because total gastrectomy is often accompanied by body mass loss and nutrient absorption disorders, such as severe hypoproteinemia and anemia, Proximal gastrectomy is more frequently recommended by researchers for early upper gastric cancer (T1N0M0) and Siewert II gastroesophageal junction cancer less than 4 cm in length. Although some functions of the stomach are retained after proximal gastrectomy, the anatomical structure of the gastroesophageal junction can be destroyed, and the anti-reflux effect of the cardia is lost. In recent years, as various reconstruction methods for anti-reflux function have been developed, some functions of the stomach are retained, and serious reflux esophagitis is avoided after proximal gastrectomy. In this article, we summarized the indications, advantages, and disadvantages of various classic reconstruction methods and latest improved reconstruction method including esophageal and residual stomach anastomosis, tubular gastroesophageal anastomosis, muscle flap anastomosis, jejunal interposition, and double-tract reconstruction.

## Introduction

Gastric cancer is one of the most common malignant tumors globally. In 2018, gastric cancer ranked fifth and third among malignant tumors in global incidence and mortality, respectively. China accounted for 44.1% and 49.9% of the global incidence and mortality of gastric cancer, respectively^1^. National Cancer Center data in 2020 revealed that gastric cancer is the second and third most common cancer in men and women, respectively, and the third leading cause of cancer-related mortality^2^. Over the past 30-40 years, the incidence of gastroesophageal junction adenocarcinoma has gradually increased^3^,^4^,^5^. Proximal gastrectomy (PG) or total gastrectomy is recommended for early gastric cancer of the upper third of the stomach^6^,^7^. Because total gastrectomy is often accompanied by body mass loss and nutrient absorption disorders, such as severe hypoproteinemia and anemia, PG is more frequently recommended by researchers for early upper gastric cancer (T1N0M0) and Siewert II gastroesophageal junction cancer less than 4 cm in length^8^,^9^,^10^,^11^,^12^. Although some functions of the stomach are retained after PG, the anatomical structure of the gastroesophageal junction can be destroyed, and the anti-reflux effect of the cardia is lost. The retained pylorus delays gastric emptying to a certain extent^13^. Severe reflux esophagitis and anastomotic stenosis are common after PG^14^. In recent years, as various reconstruction methods for anti-reflux function have been developed, some functions of the stomach are retained, and serious reflux esophagitis is avoided after PG. Although there are many reviews on digestive tract reconstruction after proximal gastrectomy, this manuscrpit adds new research progress in the last two years. In this article, the indications, advantages, and disadvantages of various reconstruction methods are summarized.

## Esophageal and residual stomach anastomosis

### Esophageal and residual stomach anastomosis

**Figure 1:**
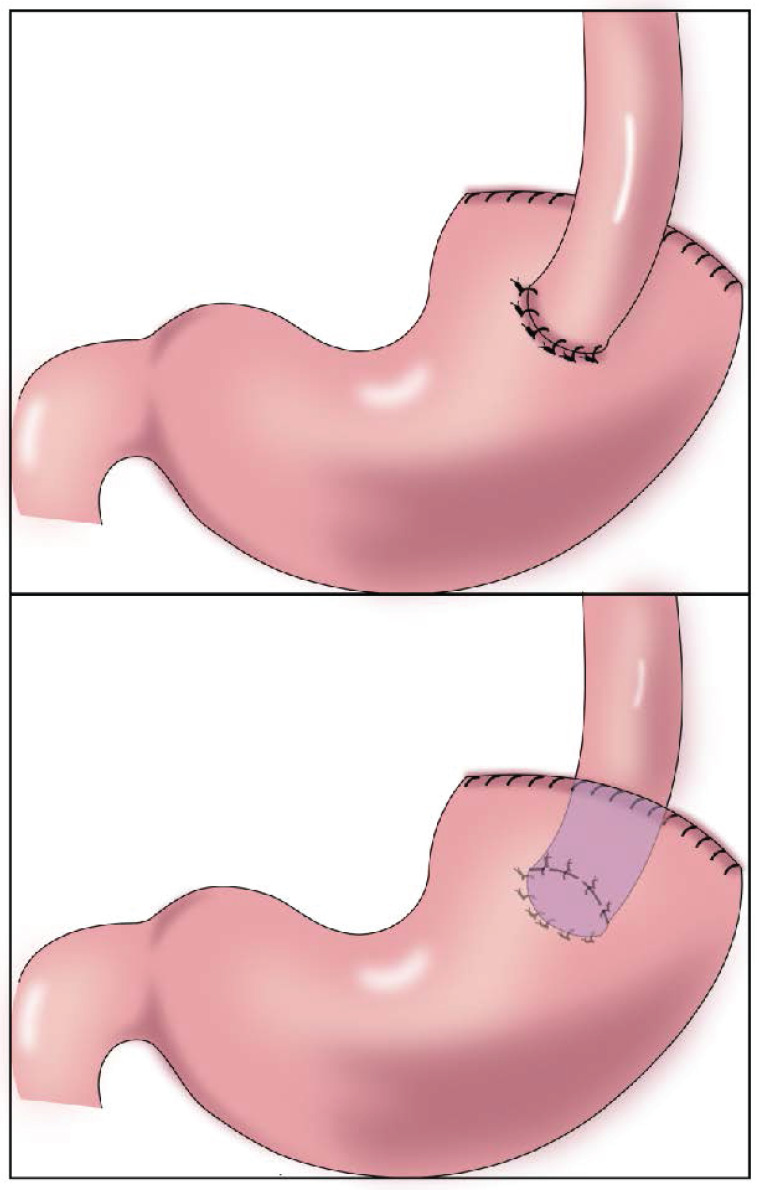
Gastroesophagostomy: The esophagus was anastomosed with the anterior wall of the residual stomach after proximal gastrectomy (above), the esophagus was anastomosed with the posterior wall of the residual stomach after proximal gastrectomy (below).

In 1897, Mikulicz performed esophageal and residual stomach anastomosis for the first time (Figure 1)^15^. This procedure resulted in a high incidence of postoperative complications (such as reflux esophagitis, abnormal gastric emptying, and malnutrition), but it was later improved to esophagogastric anterior wall anastomosis (Figure 1). The top of the retained stomach forms a structure similar to the stomach bottom, creating an angle of His, with a certain anti-reflux effect^13^. Esophageal and gastric anastomosis conforms to the physiological structure of the digestive tract, ensuring that the residual stomach has sufficient digestion and absorption function. After chyme is digested through the residual stomach and passes through the duodenum, the secretion of bile and pancreatic juice can be promoted, thereby promoting the digestion and absorption of food. Simple esophageal and residual stomach anastomosis causes a high incidence of reflux esophagitis, which seriously affects the postoperative quality of life. Reflux results in a high incidence of anastomotic stenosis, leading to reduced dietary intake and a worsened nutritional status^16^. Esophageal and residual stomach anastomosis is the simplest surgical method, but it is not the optimal technique (Table 1).

### Esophageal and residual stomach anastomosis and fundoplication

**Figure 2:**
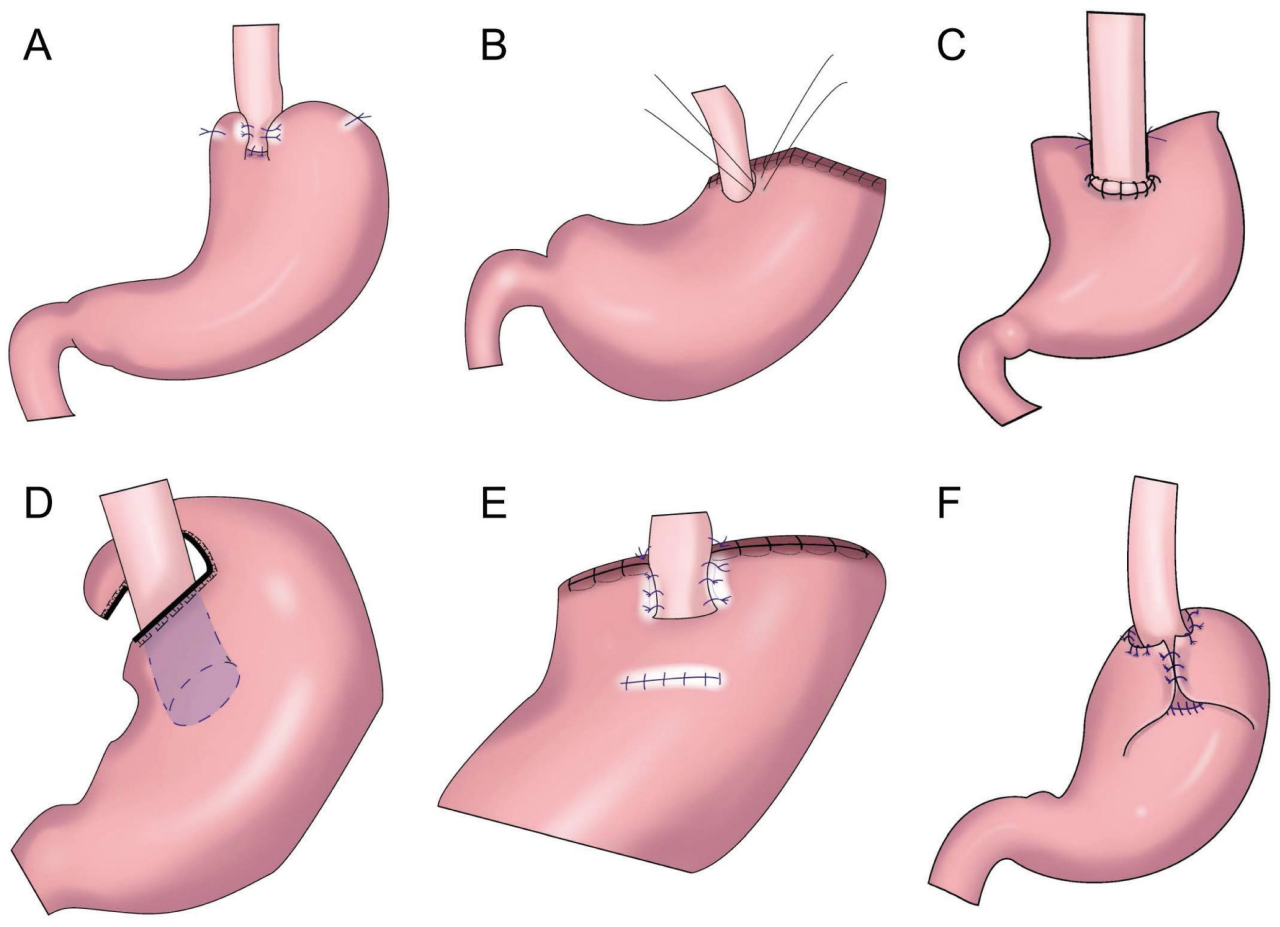
Esophagogastrostomy with posterolateral fundoplication. **A:** Toupet-like partial fundoplication (TPF), the remnant stomach was wrapped around two-thirds of the esophagus, the remnant stomach was sutured to the crura of the diaphragm. **B:** The anchoring suture created an acute angle at the anastomosis and allowed the greater curvature near the top of the remnant stomach to rebuild a new fundus. **C:** SPADE Operation, both distal part of posterior wall of esophagus and proximal part of anterior wall of stomach were fixed with two interrupted sutures, one barbed continuous suture initiated at the left corner of esophagus posterior wall and stomach anterior wall, ended on the opposite right side, anterior wall anastomosis was performed in the same maneuver. **D:** Partial neo-fundoplication, the shortened gastric remnant is wrapped around 3-quarters of the esophagus, and sutured to the distal esophagus. **E:** The cut end of the stomach was fixed to both the top posterior end of the freed esophageal wall and the diaphragm, the posterior half-circumference of the esophagus was wrapped with the anterior gastric wall by placing stay sutures. F: The residual stomach was wrapped around the anterior wall of the esophagus and sutured with 3 stitches.

To prevent reflux esophagitis, many researchers have successively performed esophageal and residual stomach anastomosis and fundoplication. Sakuramoto et al. performed laparoscopic Toupet-like partial fundoplication in 2005 (Figure 2A). One year after surgery, 15.0% of patients had heartburn symptoms and 30.0% had reflux esophagitis, but such symptoms could be controlled by proton pump inhibition^17^. In 2013, Ichikawa et al. performed esophagogastric anastomosis with a circular stapler. The anastomotic stoma was located in the anterior wall of the stomach, forming a new gastric fundus (Figure 2B). The residual stomach and lower sarcoplasmic layer of the esophagus on both sides were saturated so that the lower esophagus was surrounded by the top of the residual stomach in a semicircular manner, forming an acute angle at the esophagogastric anastomosis to prevent reflux. It was found that an acute angle at the anastomosis did not reduce reflux esophagitis^18^. In 2017, Park et al. performed laparoscopic PG with suture anchoring between the posterior wall of the esophagus and superior wall of the stomach (SPADE, Figure 2C), finding that the incidence of reflux esophagitis was 2.9%; 14.7% and 2.9% of patients had mild and moderate reflux symptoms, respectively; and the SPADE procedure could effectively reduce reflux esophagitis^19^.

Ojima et al. performed esophagogastric side-wall anastomosis with 180° fundoplication after PG, finding no reflux esophagitis 3 months after the operation via gastroscopy^20^. Polkowski et al. performed esophageal and residual stomach anastomosis after PG and fundoplication of the gastric stump around the esophagus for 3/4 (Figure 2D), observing that partial fundoplication can reduce reflux esophagitis^21^. Aizawa et al. performed PG, esophageal and residual stomach anastomosis, and posterolateral fundoplication (Figure 2E), finding that a good anti-reflux effect was achieved with posterolateral fundoplication^22^. In 2022, Zhu et al. performed laparoscopic radical gastrectomy for proximal gastric cancer, followed by “collar” type residual stomach fundoplication after esophageal and residual stomach anastomosis (Figure 2F), achieving a good anti-reflux effect after laparoscopic radical gastrectomy for proximal gastric cancer^23^.

Esophageal and residual stomach anastomosis with fundoplication can reduce the incidence of reflux esophagitis. Many fundoplication techniques with certain differences in anti-reflux effects are available. Only when the residual stomach is relatively large can esophageal and residual stomach anastomosis with fundoplication be completed (Table 1).

### Side overlap anastomosis

**Figure 3:**
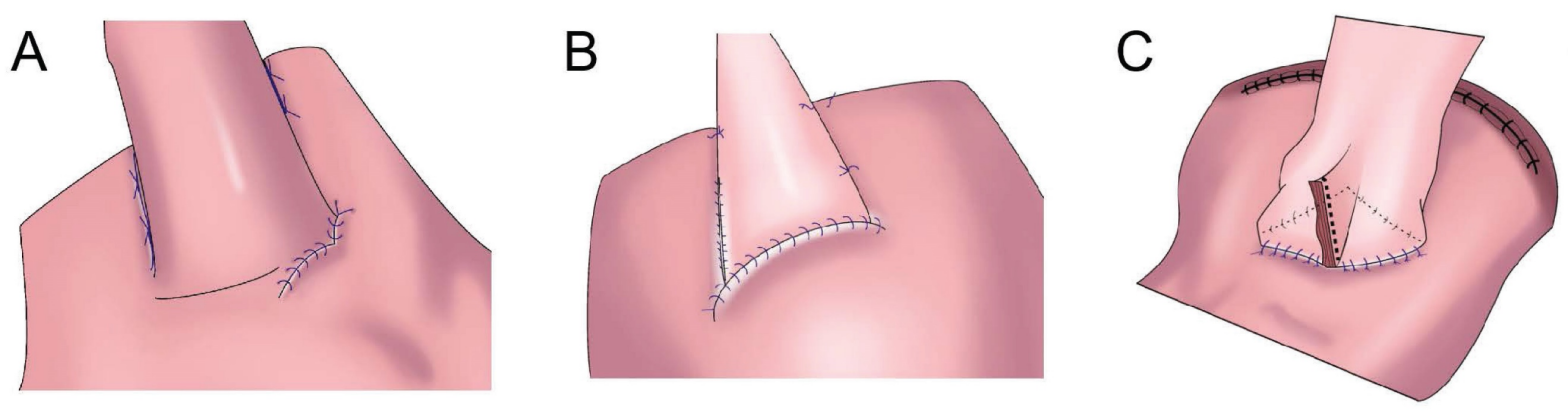
Side overlap esophagogastrostomy. **A:** After side overlap esophagogastrostomy, the linear stapler was rotated counter clockwise on its axis, suturing the gastric wall to the left side of the esophagus.** B:** After side overlap esophagogastrostomy with the right side of the esophageal stump and the anterior gastric wall, the esophagus was rotated counter clockwise on its axis, suturing the gastric wall to the left side of the esophagus, the left and lower side of the esophagus was sutured to the remnant stomach. **C:** The stapler established a connection between the esophagus and the remnant stomach without creating a common lumen to create a pseudofornix, the entry hole was closed using the laparoscopic hand-sewn suturing technique, a small V-shaped anastomosis was created.

In 2017, Yamashita et al.^24^ first reported side overlap anastomosis with fundoplication(SOFY, Figure 3A). Side overlap anastomosis generally requires that two-thirds of the abdominal esophagus and residual stomach be preserved, and the artificial gastric fundus can be reconstructed. When artificial gastric fundus pressure increases, the anastomotic stoma is closed, playing an anti-reflux role. A 60-mm linear suture device is adopted for side overlap anastomosis, resulting in a wide anastomotic orifice, thereby reducing the incidence of anastomotic stenosis^24^. The incidence of reflux esophagitis after side overlap anastomosis is 10%^25^. Yamashita et al. improved SOFY (Figure 3B) with esophageal and residual stomach anastomosis. After the operation, 17.9% of patients had reflux esophagitis in gastroscopy, but only 2.8% had reflux symptoms^26^. Fujii et al. performed laparoscopic PG and formed a “pseudo fornix” (Figure 3C) after esophageal and residual stomach anastomosis, reducing reflux esophagitis^27^.

This procedure is suitable for laparoscopy, it is relatively simple with a short anastomotic time, and it reduces the incidence of reflux esophagitis and anastomotic stenosis. However, it has the disadvantage that a long abdominal esophagus and large residual stomach (more than two-thirds) should be retained (Table 1).

## Tubular gastroesophageal anastomosis

**Figure 4:**
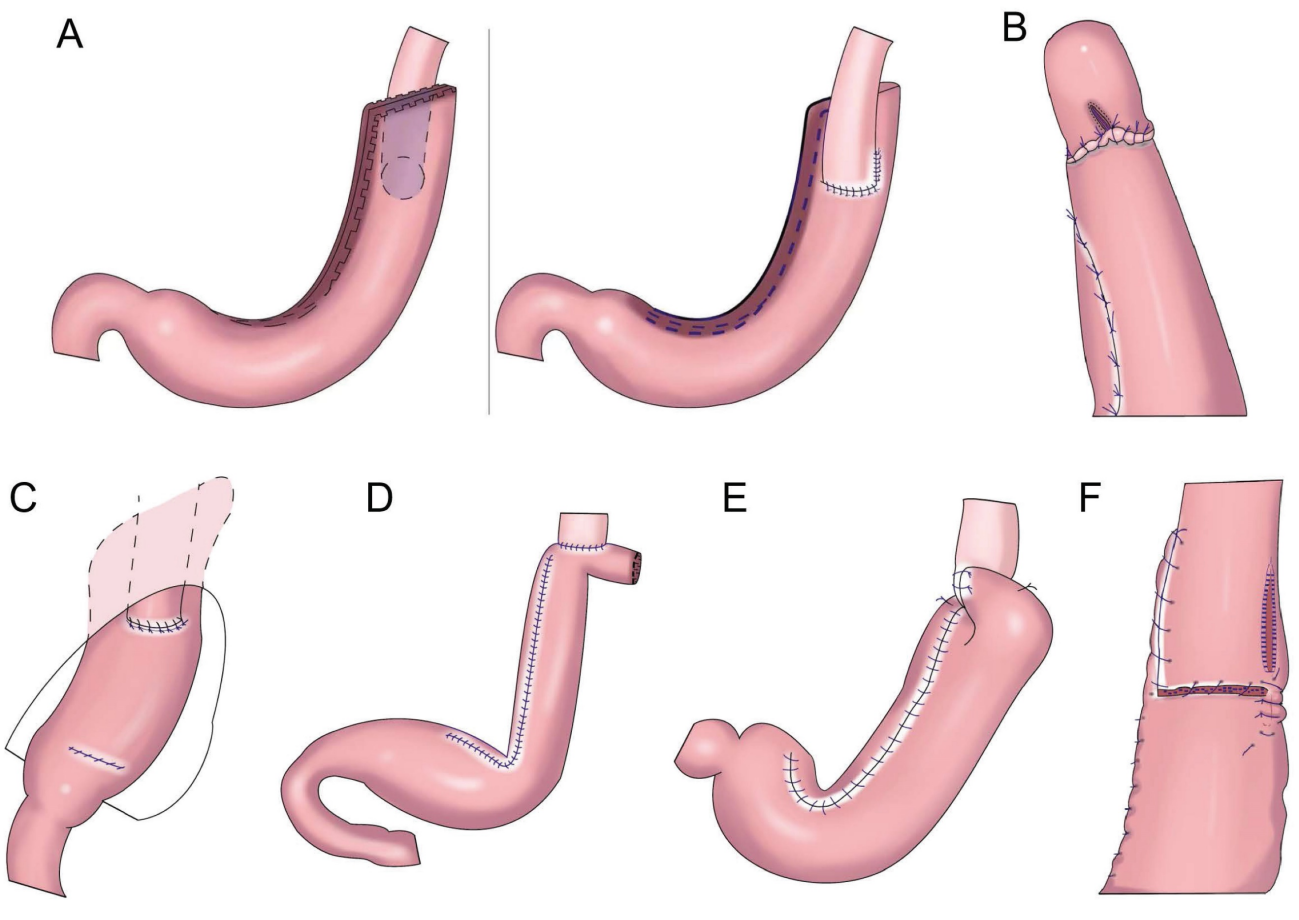
Gastric tube reconstruction. **A:** Tubular gastroesophagostomy: the esophagus was anastomosed with the anterior wall of the residual stomach(Left), the esophagus was anastomosed with the posterior wall of the residual stomach(Right), **B:** Esophagogastric tube reconstruction with stapled pseudo-fornix,** C:** A newly modified esophagogastrostomy with a reliable angle of His by placing a gastric tube in the lower mediastinum, **D:** Cheng's Giraffe reconstruction, **E:** The posterior wall of esophagus was anastomosed with the anterior wall of the residual stomach, with 180 degrees Fundoplication(the wall of the gastric tube was wrapped to the anterior aspect of the esophagus and secured to the right margin of the esophagus with two or three sutures), **F:** Side-overlap esophagogastric tube (SO-EG) reconstruction.

Shiraishi et al.^28^ first reported tubular gastroesophageal anastomosis in 1998 (Figure 4A). This operation forms a structure similar to the gastric fundus at the top of the residual stomach. When the patient is lying flat, the reflux gastric juice is temporarily stored in the “gastric fundus” to avoid direct reflux to the lower end of the esophagus to a certain extent^29^. Chen et al.^30^ found that only 14.3% of patients with tubular gastroesophageal anastomosis had postoperative reflux symptoms and that 5.7% were diagnosed with reflux esophagitis, and the degree of reflux esophagitis after tubular gastroesophageal anastomosis was lower than that after conventional esophageal and residual stomach anastomosis. Ronellenfitsch al et.^31^ found that 30% of patients had reflux symptoms after tubular gastroesophageal anastomosis, albeit with mild symptoms. Kukar et al. reported six cases of tubular stomach (posterior wall) esophageal anastomosis (Figure 4A), including two cases with anastomotic stenosis and one case with severe reflux^32^. In 2014, Hosogi et al. reported tubular stomach and pseudodome anastomosis (Figure 4B), in which the incidence of reflux esophagitis on endoscopic examination 1 year after surgery was 26.7%^33^. In 2015, Yasuda et al. inserted the upper part of the tubular stoma into the mediastinum (Figure 4C) to form an angle of His, thereby exerting an anti-reflux effect^34^. In 2018, Cheng et al. reported Giraffe reconstruction (Figure 4D), this method reconstructed the angle of His and fundus of the stomach, producing good gastric motility and anti-reflux effects according to the anatomical characteristics of the stomach and the anti-reflux mechanism of the interposed jejunum and tubular stomach^35^. Subsequently, the clinical effect of Giraffe reconstruction for PG in 100 patients with gastroesophageal junction adenocarcinoma was reported in a retrospective multicenter study. In the study, 8.0% of the patients had reflux symptoms, and 11.0% had reflux esophagitis on gastroscopy. Reflux symptoms could be controlled in all patients through behavioral guidance or oral proton pump inhibitor therapy^36^, ^37^. Good effects on reflux and gastric emptying in digestive tract reconstruction were achieved in Giraffe anastomosis, but the risk of anastomotic leak was increased because of the long narrow tubular stomach. Toyomasu et al.^38^ performed esophageal and posterior wall anastomosis and residual stomach anterior wall anastomosis with 180° esophageal folding (Figure 4E), finding that the incidence of reflux symptoms after tubular gastroesophageal anastomosis was 16.7% and that 9.8% of patients had anastomotic stenosis. Hosogi et al. revealed that the incidence of reflux esophagitis and anastomotic stricture could be reduced by esophagogastric side wall anastomosis and esophageal folding (Figure 4F)^39^.

Tubular gastroesophageal anastomosis forms a structure similar to the gastric fundus at the top of the residual stomach, avoiding direct reflux to the lower end of the esophagus to a certain extent. Part of the gastric antrum is resected for the tubular stomach, thus reducing the secretion of gastrin and gastric acid, maintaining the anatomical structure of the stomach, and improving the quality of life of patients, but the incidence of reflux esophagitis remained higher (Table 2).

## Muscle flap anastomosis

### Double-flap anastomosis (Kamikawa method)

**Figure 5:**
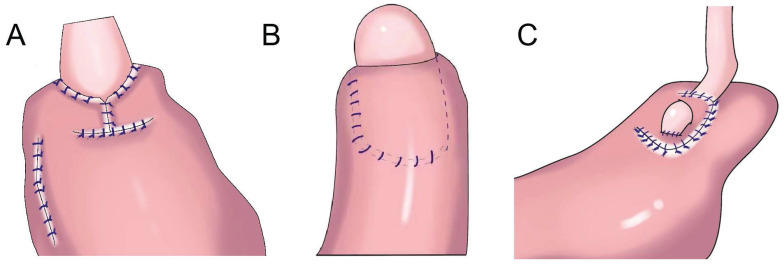
Flap anastomosis. **A:** An “エ-shaped” plasma muscle flap is made below the cutting edge of the residual stomach, the mucosa and submucosa are cut at the lower edge of the “window.” The esophageal cutting edge is anastomosed with the mucosa and submucosa, the two plasma muscle flaps are covered at the lower segment of the esophagus and upper layer of the anastomosis. **B:** "arch bridge" reconstruction of esophageal remnant stomach, the “ㄈ”-shaped single flap with an opening toward the small curved side was made, the full layer of the anterior wall of the residual stomach was cut transversely, the surrounding of the opening gastric wall was sutured, and the longitudinal opening of the single flap was closed to complete the “arch bridge”, the esophagus was pulled through the “arch bridge” of the flap of the anterior wall of the residual stomach, and the broken end of the esophagus was sutured with the opening of the anterior wall of the residual stomach.** C:** A“⊐”-shaped single flap with an opening toward the small curved side was made on the front wall of the gastric stump, the entire layer of the front wall of the gastric stump was cut at the lower edge of the muscle flap transversely, mucosal-esophageal anastomosis of the anterior wall of the stomach was performed, and the single flap and anterior wall of the stomach were continuously sutured before the anastomosis.

In 1998, Kamikawa designed the double-flap technique, also known as the Kamikawa method (Figure 5A), to prevent reflux^40^. In this operation, an “エ-shaped” plasma muscle flap is made below the cutting edge of the residual stomach, and then the mucosa and submucosa are cut at the lower edge of the “window.” The esophageal cutting edge is anastomosed with the mucosa and submucosa, and finally, the two plasma muscle flaps are covered at the lower segment of the esophagus and upper layer of the anastomosis. This method increases the pressure at the lower end of the esophagus, and it is conducive to reducing the incidence of reflux esophagitis. Kuroda et al. conducted a multicenter retrospective study to evaluate the effectiveness and safety of double-muscle valve anastomosis. The study included 546 patients from 18 centers, of whom 464 patients underwent endoscopic examination to evaluate reflux esophagitis 1 year after the surgery. The researchers found that the incidence of reflux esophagitis above grade B on endoscopy was 6%, and the incidence of anastomotic stenosis was 5.5%^41^. Double muscle flap anastomosis has a good anti-reflux effect, and it can improve the nutritional status and quality of life of patients after surgery^42^,^43^. Yamamoto et al. improved double-flap gastroesophageal reconstruction after laparoscopic PG, which can reduce reflux symptoms and the incidence of reflux esophagitis^9^. Double muscle flap anastomosis has become one of the most recommended digestive tract reconstruction methods after PG^6^, ^7^, ^41^, ^44^. Double-flap anastomosis is applicable to early gastric cancer of the upper third of the stomach with estimated residual gastric volume > 50%, but it is not applicable for patients with tumors invading the lower esophageal segment. Double-flap anastomosis can significantly reduce the incidence of postoperative hiccup and reflux esophagitis, but the technique is limited by complicated procedures, a long operative time, and a high incidence of postoperative anastomotic stenosis. Ensuring good blood supply of the double muscle flap, controlling tension, and avoiding anastomotic stenosis are the difficulties of this surgical method, which has high technical requirements for the operator and team^45^. Appropriate extension of plasma muscle flap can reduce the incidence of anastomotic stenosis^46^.

### Single-flap anastomosis

To overcome the shortcomings of complicated double-flap anastomosis, difficult surgical procedures, long operative times, and high rates of postoperative anastomotic stenosis, Peng et al. performed right open single-flap anastomosis after laparoscopic PG, finding that single-flap anastomosis had a simple procedure^47^. Li et al. performed the “arch bridge” reconstruction of the esophagus and residual stomach after PG. In vitro, the gastric wall was cut approximately 1 cm from the proximal broken end of the anterior wall of the residual stomach, and the “ㄈ”-shaped single flap with an opening toward the small curved side was made. The full layer (approximately 2 cm long) of the anterior wall of the residual stomach was cut transversely at approximately 1 cm from the lower edge of the flap, the surrounding of the opening gastric wall was sutured with 4-0 absorbable sutures, and the longitudinal opening of the single flap was closed with 4-0 absorbable sutures to complete the “arch bridge” (Figure 5B). The residual stomach was lifted up to the hole, the esophagus was pulled through the “arch bridge” of the flap of the anterior wall of the residual stomach, and the broken end of the esophagus was sutured with the opening of the anterior wall of the residual stomach. In vitro flap anchoring avoids the limitation of vision caused by laparoscopicsuture and reduces technical difficulty, and it is conducive to ensuring tunnel quality^48^. Wang et al. reported the short-term efficacy of laparoscopic PG and esophagogastrostomy with single-flap technology in seven patients, observing satisfactory short-term efficacy for laparoscopic PG with single-flap anastomosis^49^. Yang et al.^50^ proposed single-flap anastomosis on the basis of double-flap anastomosis, that is, a “⊐”-shaped single flap with an opening toward the small curved side was made on the front wall of the gastric stump, the entire layer of the front wall of the gastric stump was cut at the lower edge of the muscle flap transversely, mucosal-esophageal anastomosis of the anterior wall of the stomach was performed, and the single flap and anterior wall of the stomach were continuously sutured before the anastomosis. A multicenter, prospective, randomized controlled study with a target completion date of July 31, 2027 could provide better conclusions from the research results.

The anastomotic stoma of the esophagus and stomach with a muscle flap is wrapped into a soft valve that acts as a one-way valve with good anti-reflux effects. The anastomotic stoma was wrapped with a seromuscular layer, resulting in a low incidence of anastomotic leak. However, muscle flap anastomosis has a complicated procedure, high requirements for suture technology, and a long operative time, thus increasing the incidence of anastomotic stenosis (Table 3).

## Jejunal interposition

**Figure 6:**
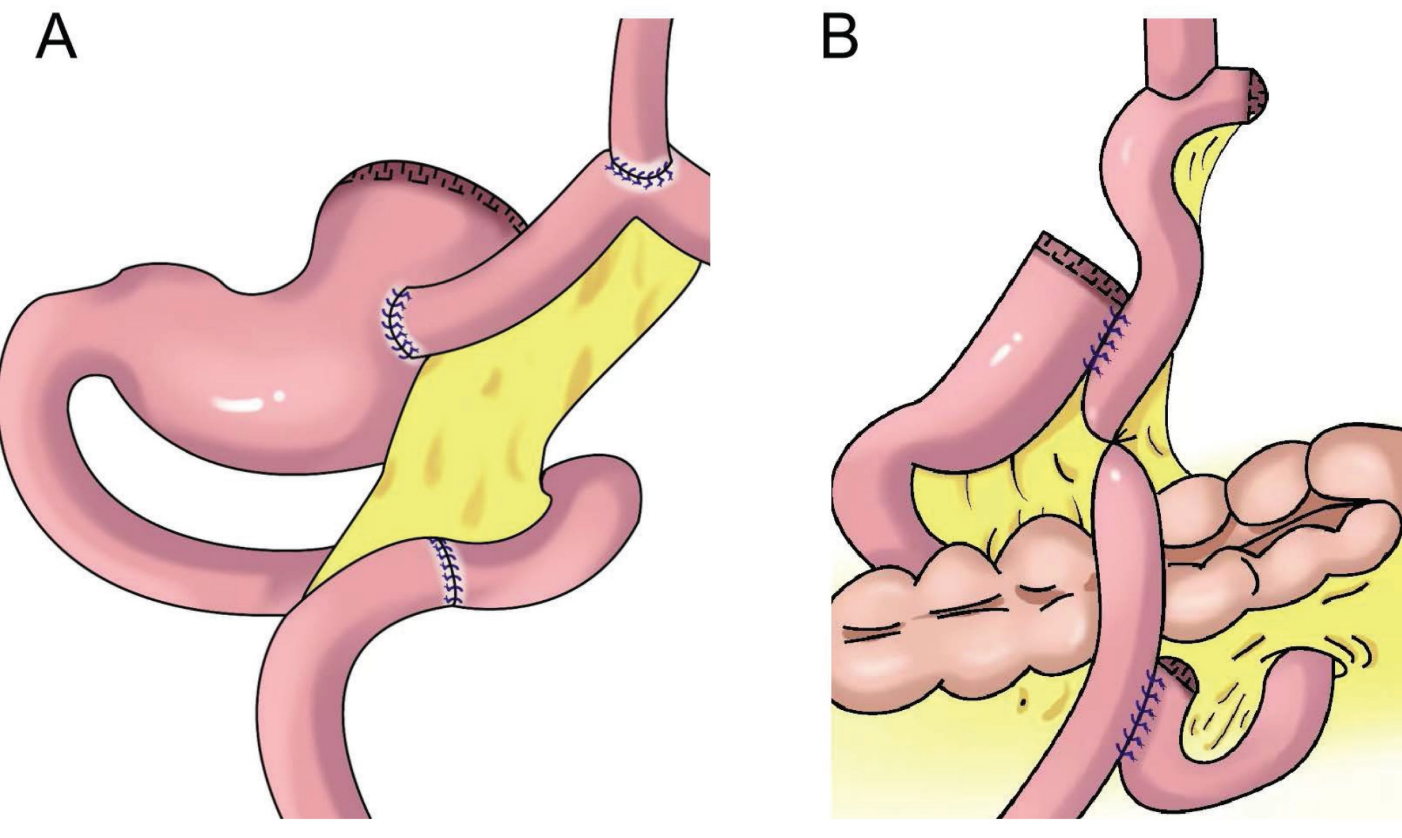
Jejunal interposition reconstruction. **A:** jejunal interposition reconstruction, between the residual stomach and esophagus, inserting a section of jejunum approximately 10-15 cm. **B:** piggyback jejunal interposition reconstmction with uncut jejunal continuity.

Jejunal interposition aims to restore the connectivity between the esophagus and residual stomach by inserting a section of jejunum between the esophagus and residual stomach and build an anti-reflux barrier between the residual stomach and esophagus using the tolerance of the jejunum itself to acidic gastric juice and alkaline intestinal juice and the natural peristalsis of the intestine. In 1941, Zhenxin^51^ first reported jejunal interposition (Figure 6A). In the 1960s, short jejunal interposition and pylorus formation were generally performed. In the 1970s, to better resist reflux, the length of the interposed jejunum was up to 30-40 cm. An excessive length of jejunal interposition was not conducive to endoscopy, and it increased the retention time of food in the intestine^52^. At present, the length of jejunal interposition tends to be approximately 10-15 cm^53^,^54^. Kameyama et al.^55^ reported that jejunal interposition + storage bag insertion can retain the storage capacity, and it is easy to observe the curative effect through endoscopy after operation. Katai et al. found that the incidence of reflux after jejunal interposition was 5.5%, and the incidence of endoscopic reflux esophagitis was 1.7%^56^. Jejunal interposition can ensure the scope of gastrectomy, preserve pyloric function and the physiological channel of food, effectively resist reflux, and improve the quality of life of patients after surgery^57^. The upper part of the stomach is replaced by the small intestine. Compared with the stomach, the fascia of the small intestine is thinner, and its storage capacity is limited by hyphological weakness. Xu reported PG combined with jejunum interposition. Based on double-tract reconstruction, jejunal access was blocked at the distal end of the gastrojejunostomy(Figure 6B)., preserving the continuity of the interposition jejunal bowel, reducing the possibility of food emptying disorders, and improving the nutritional status of patients^58^, ^59^, ^60^.

This method has low requirements concerning the size of the residual stomach, and it is suitable for reconstruction after most PG procedures. However, because of the complicated operative procedure, long operative time, and high cost, the possibility of emptying obstruction and internal hernia increases^61^.

## Double-tract reconstruction

In 1988, Aikou et al.^62^ first reported PG with double-tract reconstruction in which the proximal stomach was severed, followed by esophagojejunal Roux⁃en⁃Y anastomosis and side overlap between the residual stomach and esophagojejunal anastomosis 10-15 cm distal to the jejunum. This strategy allowed food to enter the distal jejunum through both the residual stomach and jejunum after esophagojejunostomy. However, with double-tract reconstruction, food often cannot enter the duodenum smoothly.

**Figure 7:**
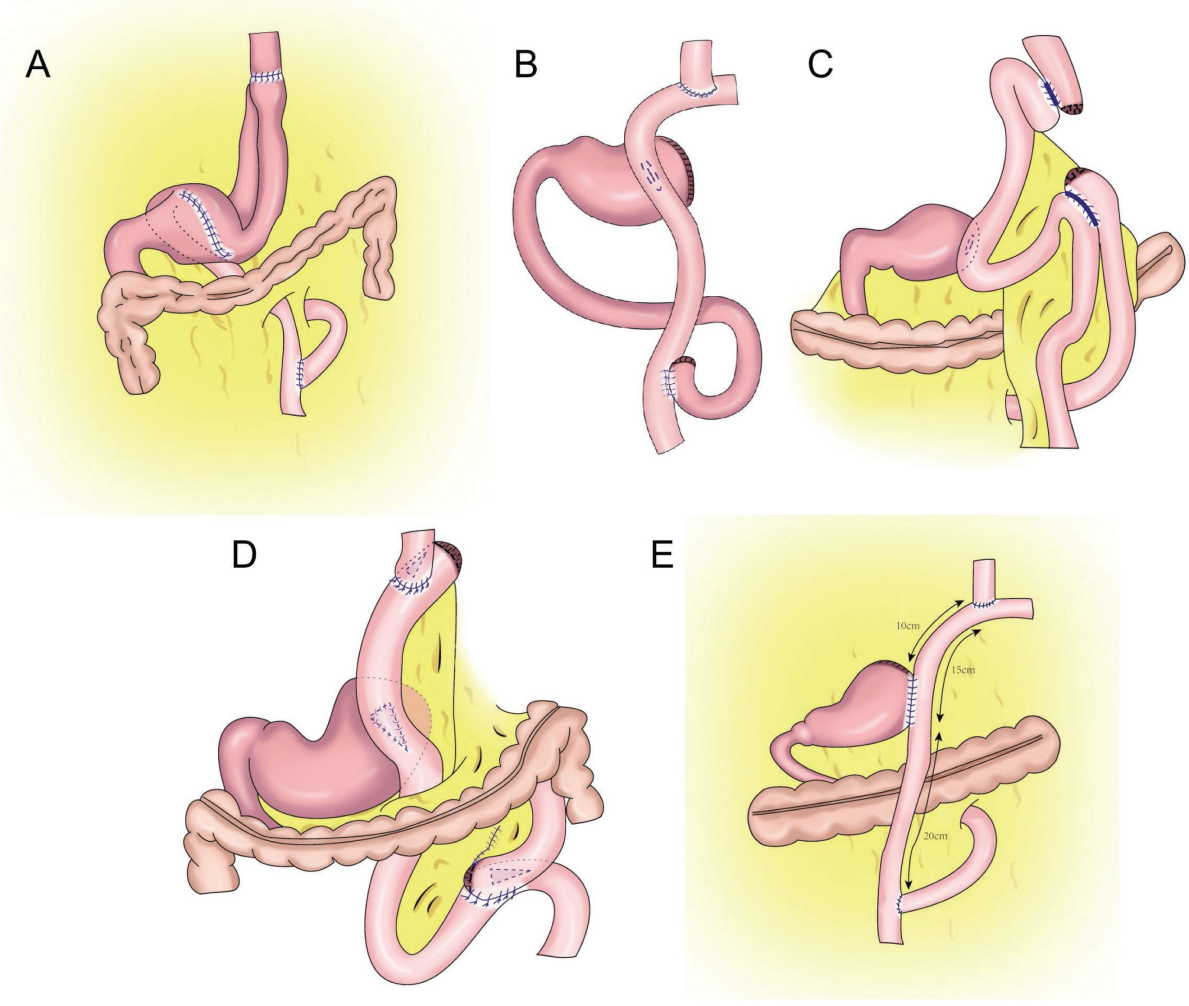
Double tract reconstruction. **A:** N-shaped double tract method, the residual stomach is twisted anteriorially by 180 degrees, the seromuscular sutures are inserted to reinforce the posterior wall before restoring the twist, after the gastrojejunostomy, the twisting of the residual stomach is restored to its usual portion. **B:** Bouble tract reconstruction of the remnant stomach anterior wall. **C:** Oblique jejunogastrostomy method (OJG), the jejunum is transected at a point 20-cm distant from the ligament of Treitz, a side-to-side esophagojejunostomy is performed, the stomach and jejunum are twisted posteriorly, and the posterior wall of the remnant stomach and the posterior wall of the jejunum are put together. An oblique side-to-side jejunogastrostomy from the antimesenteric wall to the posterior wall is performed, the jejunum returns the torsion of the jejunum to the counter clockwise direction and rides on the remnant stomach, a side-to-side jejunojejunostomy is made between the jejunum 20 cm below the jejunogastrostomy and the proximal jejunum. **D:** double tract reconstruction of colon posterior. **E:** double tract reconstruction of the remnant stomach side wall.

Later, Aikou et al. improved the traditional double-tract reconstruction technique. Namely, after PG, esophagojejunostomy is performed behind the colon and residual stomach, and jejunostomy is performed at a distance of approximately 40 cm from the distal end of the anastomosis. The residual stomach is rotated 180° before anastomosis and restored to its normal position after anastomosis. The jejunum is “N”-shaped around the gastrojejunal anastomosis (Figure 7A), allowing food to enter the residual stomach more easily, resulting in a good anti-reflux effect^62^. In theory, double-tract reconstruction is a relatively ideal reconstruction method. Because food enters the distal digestive tract through two tracks, the residual stomach has the function of storing and digesting food, and some food can pass through the duodenum, thus extending the time food remains in the digestive tract and allowing food and digestive fluid to more fully mix for better nutrient absorption. At the same time, expansion of the residual stomach after eating can also increase the appetite of patients^63^,^64^,^65^. The retention of food in the residual stomach can induce the secretion of gastrin, facilitating the balance of gastrointestinal hormones, reducing the incidence of dumping syndrome, and increasing the absorption of iron and vitamin B^66^,^67^. Double-tract reconstruction can effectively reduce gastroesophageal reflux, and the incidence of reflux esophagitis is approximately 8.0%-13.8%^68^,^69^,^70^,^71^,^72^. Food sometimes cannot be evacuated according to the theoretical designed double-tract reconstruction, but most food directly enters the jejunum^73^. To avoid such a situation, domestic and foreign experts have improved the size and direction of gastrojejunal anastomosis to allow more food to enter the residual stomach^65^,^74^ (Figure 7B-E). Xu et al. used a 60-mm linear cutting occluder to anastomose the anterior wall of the residual stomach with the jejunum to widen the anastomosis, which is theoretically more conducive to food entering the residual stomach^75^. Sato et al. performed laparoscopic PG with postcolonic double-tract reconstruction, finding that double-tract reconstruction was safe and feasible and was significantly better than total gastrectomy in terms of quality of life and nutritional status^76^.

Double-tract reconstruction is applicable to reconstruction of the digestive tract after most PGs. It has low requirements concerning the volume of the residual stomach, and it is applicable to patients who have undergone an excessive number of gastrectomies and who are not suitable for esophageal and residual stomach anastomosis. However, this surgery is relatively complicated and expensive, and it results in many anastomoses, which might increase the risk of anastomotic leak.

## Conclusion

Since the development of digestive tract reconstruction after PG, various improved methods have been introduced. Each reconstruction method is constantly changing and improving. Each reconstruction method has its own advantages and disadvantages. Although no uniform standard is available at present, in clinical practice, all surgeons have their own unique experience and thinking regarding the available reconstruction methods. Understanding the operating points and advantages and disadvantages of various digestive tract reconstruction can provide ideas for surgeons to make correct clinical decisions.

## Figures and Tables

**Table 1:** Anti-reflux effect of gastroesophagostomy after proximal gastrectomy for gastric cancer

Author	Anastomotic method	Time of first report	Disadvantage
Only Gastroesophagostomy			Esophageal and residual stomach anastomosis is the simplest surgical method, high incidence of reflux esophagitis and anastomotic stenosis was higher.
Mikulicz^15^	Gastroesophagostomy	1897	
Gastroesophagostomy with Fundoplication			Esophageal and residual stomach anastomosis with fundoplication can reduce the incidence of reflux esophagitis. Many fundoplication techniques with certain differences in anti-reflux effects are available. Only when the residual stomach is relatively large can esophageal and residual stomach anastomosis with fundoplication be completed.
Sakuramoto^17^	laparoscopy-assisted proximal gastrectomy with Toupet-like partial fundoplication	2009	
Ichikawa^18^	Esophagogastrostomy with anchoring suture created an acute angle at the anastomosis and frebuilding a new fundus	2013	
Park^19^	SPADE Operation	2017	
Ojima^20^	Gastroesophagostomy with 180 degrees Fundoplication (the remnant stomach was wrapped from the esophageal posterior wall towards the esophageal anterior wall.	2018	
Polkowski^21^	Posterior Esophago-Gastrostomy and Partial Neo-Fundoplication	2020	
Aizawa^22^	Esophagogastrostomy with Posterolateral Fundoplication	2021	
Zhu^23^	Esophagogastrostomy with “collar” fundoplication	2022	
Side overlap esophagogastrostomy			This procedure is suitable for laparoscopy, it is relatively simple with a short anastomotic time, and it reduces the incidence of reflux esophagitis and anastomotic stenosis. However, it has the disadvantage that a long abdominal esophagus and large residual stomach (more than two-thirds) should be retained.
Yamashita^24^	side overlap with fundoplication by Yamashita (SOFY)	2017	
Yamashita^26^	modified SOFY (mSOFY)	2022	
Fujii^27^	Esophagogastric Anastomosis With Stapled Pseudo-Fornix	2022	

**Table 2:** Anti-reflux effect of Gastric tube reconstruction after proximal gastrectomy for gastric cancer

Author	Anastomotic method	Time of first report	Advantages and disadvantages
Shiraishi^28^	Gastric tube reconstruction: the esophagus was anastomosed with the anterior wall of the residual stomach	1998	the incidence of reflux symptoms and anastomotic stenosis was higher
Hosogi^33^	Esophagogastric tube reconstruction with stapled pseudo-fornix	2014	With the advantage of a gastric tube, a tension-free anastomosis was possible even for bulky tumors that needed lower esophagectomy, but reflux esophagitis was higher
Yasuda^34^	A newly modified esophagogastrostomy with a reliable angle of His by placing a gastric tube in the lower mediastinum	2015	the formation of a pseudo-fornix and optimal angle of His appeared to reduce bile reflux, the gastric remnant in the form of a narrow gastric tube with low compliance resulted in decreased food residue.
Cheng^35^	Cheng's Giraffe reconstruction	2018	The reconstructed digestive tract is consistent with physiological characteristics and has good anti-reflux effects, but the risk of anastomotic leak was increased because of the long narrow tubular stomach.
Kukar^32^	Tubular gastroesophagostomy: the esophagus was anastomosed with the posterior wall of the residual stomach	2018	the incidence of anastomotic strictures, and significant reflux were higher.
Toyomasu^38^	Tubular gastroesophagostomy with 180 degrees fundoplication	2021	Tubular gastroesophagostomy with 180 degrees fundoplication has anti-reflux effects, the incidence of anastomotic strictures were higher.
Hosogi^39^	Side-overlap esophagogastric tube (SO-EG) reconstruction	2022	Fundoplication with a longer overlap might be better to completely prevent refux.

**Table 3:** Anti-reflux effect of flap anastomosis after proximal gastrectomy for gastric cancer

Author	Anastomotic method	Time of first report	Advantages and disadvantages
Kamikawa^40^	double⁃flap anastomosis	1998	complicated surgical suture technique, difficult operation, strict surgical indications and high incidence of postoperative anastomotic stenosis
Peng^47^	right-open single-flap technique	2022	Compared with double-flap anastomosis, the operation of single-flap anastomosis is relatively simple, and the blood supply at the edge of the single-flap may be worse than that of the double-flap. More clinical data are still needed to verify its safety.
Li^48^	"arch bridge" reconstruction of esophageal remnant stomach	2022	
Yang^50^	left-open single-flap technique	2022	
